# Early-onset pancreatic cancer: a population-based study using the SEER registry

**DOI:** 10.1007/s00423-019-01810-0

**Published:** 2019-08-03

**Authors:** Daniel Ansari, Carl Althini, Henrik Ohlsson, Roland Andersson

**Affiliations:** Department of Surgery, Clinical Sciences Lund, Lund University and Skåne University Hospital, SE-221 85 Lund, Sweden

**Keywords:** Early-onset pancreatic cancer, EOPC, Population-based study, Propensity score matching, SEER

## Abstract

**Background:**

Early-onset pancreatic cancer (< 50 years, EOPC) is uncommon and limited data exist on clinical presentation and long-term survival. The aim of this study was to compare outcomes between patients with EOPC and those with later-onset pancreatic cancer (≥ 50 years, LOPC) using a large population-based cohort.

**Methods:**

The Surveillance, Epidemiology, and End Results (SEER) database was queried to identify patients with a microscopically confirmed pancreatic ductal adenocarcinoma for the period 2004 to 2016. Propensity score matching was used to compare overall survival (OS) and cancer-specific survival (CSS) between patients with EOPC and LOPC. The EOPC and LOPC patients were paired 1:1 on propensity scores based on gender, tumor location, tumor size, AJCC stage, and treatment details.

**Results:**

The overall cohort included 72,906 patients with pancreatic ductal adenocarcinoma, including 4523 patients with EOPC (6.2%). EOPC patients were diagnosed at a more advanced AJCC stage (*p* < 0.001) compared with LOPC patients and received significantly more treatment, including surgery (*p* < 0.001), radiation (*p* < 0.001), and chemotherapy (*p* < 0.001). Following propensity score matching, 3172 EOPC patients were matched to 3172 LOPC patients, alleviating any covariate differences between the groups. The matched analysis showed that EOPC was associated with poorer 5-year OS (6.1% vs 8.6%, *p* = 0.003) and 5-year CSS (6.7% vs 9.7%, *p* < 0.001). In multivariable Cox regression analysis, EOPC remained significantly associated with adverse OS and CSS. Subgroup analyses showed that EOPC was associated with adverse 5-year OS (17.7% vs 26.9%, *p* < 0.001) and 5-year CSS (18.9% vs 29.7%, *p* < 0.001) in operated patients. After multivariable analysis, EOPC remained significantly associated with OS and CSS. For patients that did not undergo surgery, the OS and CSS remained dismal without any significant differences between the groups.

**Conclusion:**

To our knowledge, this is the largest study to compare the outcome of EOPC vs LOPC, as well as the first to use propensity score matching methodology for this purpose. The findings demonstrate that EOPC is diagnosed at a later stage and the matched survival analysis demonstrated reduced OS and CSS. We suggest that pancreatic cancer in young patients may have a unique tumor biology, which may be of importance for risk stratification and patient counseling.

## Introduction

Pancreatic cancer is a severe type of cancer with an overall 5-year survival in the single digits [1]. Although pancreatic cancer is the 11th most common cancer type, it ranks as the third leading cause of cancer-related death in the USA [2]. The incidence of pancreatic cancer is also increasing [3]. By the year 2030, pancreatic cancer is projected to become the second leading cause of cancer-related death [4]. The main reasons for the poor outcome of pancreatic cancer are delayed diagnosis, aggressive tumor phenotype, and treatment resistance.

Most patients with pancreatic cancer are in the 60–80-year age group [1, 3, 5, 6]. Pancreatic cancer is rare before the age of 50 and these patients are referred to as having early-onset pancreatic cancer (EOPC) [7–9]. Although a small fraction, the EOPC group greatly contributes to the societal burden of pancreatic cancer, with a high number of potential years of life lost (PYLL).

Raimondi et al. [10] attributed 25% of PYLL in pancreatic cancer in the USA as a direct cause of EOPC. In several European countries, this rate may be as high as 40%. There have been efforts to identify risk groups and risk factors for developing EOPC. Cigarette smoking has been proposed as the most important risk factor for EOPC [7, 10]. In addition, high intake of alcohol, genetic disorders, and familiar pancreatic cancer have also been suggested to contribute to the development of EOPC [11].

It is still not established whether or not EOPC differs from later-onset pancreatic cancer (LOPC) in terms of tumor biology. Two previous studies have compared molecular features between patients < 40 years and their older counterparts [12, 13]. These studies have shown an overlap in molecular biology between EOPC and LOPC. However, the studies have also shown several distinctive features in young patients, including low rates of KRAS mutations, suggesting the presence of still undefined tumor-initiating events in this subgroup.

Early-onset breast cancer in women has been shown to behave more aggressively and more often present at an advanced stage than later-onset breast cancer [14]. We hypothesize that the same might be true for pancreatic cancer. The objective of this study was to determine whether the clinical features, overall survival (OS), and cancer-specific survival (CSS) of EOPC differ from those in older patients by using population-based data from a large cohort.

## Methods

### Data source

The data were obtained from the National Cancer Institute Surveillance, Epidemiology, and End Results (SEER) database (SEER*Stat version 8.3.5) for the years 2004 to 2016. The SEER registry collects cancer incidence and survival data from population-based cancer registries covering approximately 34.6% of the US population.

### Patients and study design

One hundred forty-three thousand, one hundred fifty-seven patients were identified on the basis of the International Classification of Diseases for Oncology, 3rd edition (ICD-O-3) for tumors of the exocrine pancreas: C25.0, C25.1, C25.2, C25.3, C25.7, C25.8, and C25.9. Out of these patients, 51,811 were excluded from the study due to not having a histologically or cytologically confirmed pancreatic adenocarcinoma or ductal adenocarcinoma of the pancreas (ICD-O-3 histology codes 8140 and 8500, respectively). An additional 18,440 patients were excluded due to lack of outcome or follow-up data. The final study cohort included 72,906 patients. The following parameters were extracted: age, gender, tumor size, tumor location, clinical stage, type of treatment (cancer-directed surgery, radiation, chemotherapy), and survival. Histological grade was not evaluated in this study due to the high amount of missing data (64% missing values). The study followed the STROBE guidelines [15], where applicable.

### Definitions

In this study, EOPC was defined as disease occurring in patients younger than 50 years, in accordance with previous definitions [7–9, 16]. Those who were diagnosed ≥ 50 years were defined as having LOPC. The American Joint Committee on Cancer (AJCC) 7th edition staging system was used to classify tumors throughout the study period. OS was defined as the time interval from diagnosis to death from any cause or date of last follow-up. The SEER cause-specific death classification variable was used to obtain CSS.

### Propensity score matching

Propensity score matching offers a method to reduce bias in observational studies where randomized treatment assignment is not possible. We calculated individual propensity scores through logistic regression modeling based on the following 7 covariates: gender, tumor size, tumor location, AJCC stage, surgery, radiation, and chemotherapy. The EOPC and LOPC patients were then paired 1:1 on these propensity scores using exact matching.

### Statistical analysis

The data were analyzed using SPSS Statistics 25 (IBM Corp., Armonk, NY, USA) and Stata MP 14.1 software (StataCorp LP, College Station, TX, USA). Categorical data are presented as frequencies with percentage and continuous data are expressed as median with range. Baseline characteristics between age groups were compared using Mann-Whitney *U* tests for continuous variables and the chi-square tests for categorical variables. OS and CSS were calculated for the matched patients and modeled using the Kaplan–Meier method. Statistical differences between the survival curves were assessed with the log-rank test. We estimated the hazard ratios (HRs) and 95% confidence intervals (CIs) for the association between EOPC and survival independent of other risk factors by fitting a multivariable Cox proportional hazard regression model. Any variable from the univariable test with a *p* value < 0.25 was selected as a candidate for the multivariable analysis. In the iterative process of variable selection, covariates were removed from the model if they were non-significant and not a confounder, as described by Hosmer-Lemeshow [17], resulting in a main effect model.

## Results

**Fig. 1:**
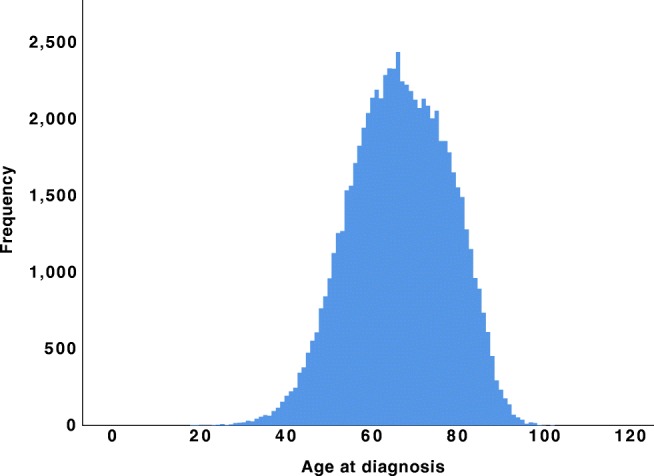
Age distribution for the entire cohort (*N* = 72,906)

A total of 72,906 patients with pancreatic cancer were included in the study. The median age was 68 (range 19–103) years. Figure 1 shows the age distribution in the study. The cohort consisted of 35,257 women and 37,649 men. The AJCC stage at diagnosis included stage I (*N* = 3790; 6.1%), stage II (*N* = 18,005; 29.0%), stage III (*N* = 6481; 10.4%), stage IV (*N* = 33,870; 54.5%), and unknown stage (*N* = 10,760, 14.7%). The median OS was 7 months for the entire cohort, with a 5-year survival rate of 4.9%.

### Patient characteristics before propensity score matching

A total of 4523 patients were classified as having EOPC (6.2%). Demographic and clinical data from the patients with EOPC were compared with those who had LOPC (Table 1). Gender distribution was significantly different between the groups, with higher rate of male patients in the EOPC cohort (58.0% vs 51.2%, *p* < 0.001). EOPC presented with more advanced AJCC stage at diagnosis. The rate of distant metastasis (stage IV) was 58.7% in the EOPC group vs 54.2% in the LOPC group, *p* < 0.001. No significant difference could be found between EOPC and LOPC regarding tumor location and tumor size. EOPC patients received more active treatment. Surgery was offered to 23.2% in the EOPC group compared with 19.9% in the LOPC group, *p* < 0.001. Radiation therapy was also more frequently administered in the EOPC group (23.6% vs 17.6%, *p* < 0.001). Chemotherapy was given to 73.3% in the EOPC group compared with 55.4% in the LOPC group, *p* < 0.001.

**Table 1:** Baseline characteristics of the EOPC and LOPC groups

Variable	*N*	EOPC *N* = 4523	LOPC *N* = 68,383	*P* value
Age, years (range)	72,906	46 (19–49)	69 (50–103)	
Male gender	72,906	2625 (58.0%)	35,024 (51.2%)	< 0.001
Tumor location (pancreatic head)	72,906	2301 (50.9%)	35,512 (51.9%)	0.168
Tumor size > 2 cm	57,352	3275 (91.5%)	48,923 (91.0%)	0.340
AJCC stage 7th edition	62,146			< 0.001
I		157 (3.9%)	3633 (6.3%)	
II		1039 (25.9%)	16,966 (29.2%)	
III		461 (11.5%)	6020 (10.4%)	
IV		2359 (58.7%)	31,511 (54.2%)	
Surgery	72,092	1037 (23.2%)	13,456 (19.9%)	< 0.001
Radiation therapy (yes vs no*)	72,279	1058 (23.6%)	11,904 (17.6%)	< 0.001
Chemotherapy (yes vs no*)	72,906	3316 (73.3%)	37,907 (55.4%)	< 0.001

*N*, number of non-missing values. Qualitative data are expressed as *N* (%) and quantitative data as median (range). *No evidence of radiotherapy or chemotherapy was found in the medical records examined. *EOPC*, early-onset pancreatic cancer; *LOPC*, later-onset pancreatic cancer

### Characteristics of propensity score–matched cohorts

Following propensity score matching, 3172 EOPC patients were matched to 3172 LOPC patients. Previously observed covariate differences between cohorts were alleviated after matching (Table 2). Due to the large cohort size, exact matching could be achieved for every patient.

**Table 2:** Comparison of Clinical Characteristics after Propensity Score Matching

Variable	EOPC *N* = 3172	LOPC *N* = 3172	*P* value
Age, years (range)	46 (19–49)	67 (50–96)	
Male gender	1844 (58.1%)	1844 (58.1%)	1.000
Tumor location (pancreatic head)	1772 (55.9%)	1772 (55.9%)	1.000
Tumor size > 2 cm	2892 (91.2%)	2892 (91.2%)	1.000
AJCC stage 7th edition			1.000
I	147 (4.6%)	147 (4.6%)	
II	943 (29.7%)	943 (29.7%)	
III	373 (11.8%)	373 (11.8%)	
IV	1709 (53.9%)	1709 (53.9%)	
Surgery	869 (27.4%)	869 (27.4%)	1.000
Radiation therapy (yes vs no*)	855 (27.0%)	855 (27.0%)	1.000
Chemotherapy (yes vs no*)	2429 (76.6%)	2429 (76.6%)	1.000

Qualitative data are expressed as *N* (%) and quantitative data as median (range). *No evidence of radiotherapy or chemotherapy was found in the medical records examined. *EOPC*, early-onset pancreatic cancer; *LOPC*, later-onset pancreatic cancer

### Survival analyses

**Fig. 2:**
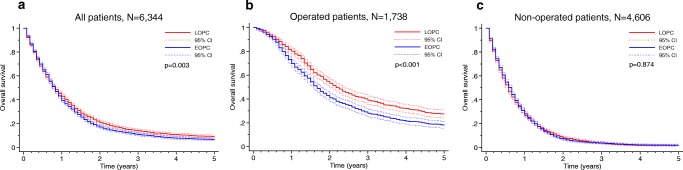
Propensity-matched comparison of overall survival between the EOPC and LOPC groups in panels **a** all patients, **b** operated patients, and **c** non-operated patients. Statistical test: log-rank test

**Fig. 3:**
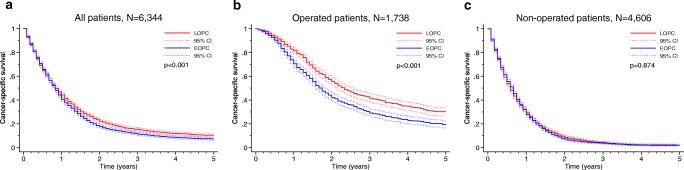
Propensity-matched comparison of cancer-specific survival between the EOPC and LOPC groups in panels **a** all patients, **b** operated patients, and **c** non-operated patients. Statistical test: log-rank test

Survival analyses in matched patients showed that EOPC was associated with poorer 5-year OS (6.1% vs 8.6%, *p* = 0.003) and 5-year CSS (6.7% vs 9.7%, *p* < 0.001), as shown in Figs. 2a and 3a, respectively. In multivariable Cox regression analysis, EOPC remained significantly associated with adverse OS and CSS (Tables 3 and 4). Subgroup analyses showed that EOPC was associated with adverse 5-year OS (17.7% vs 26.9%, *p* < 0.001) and 5-year CSS (18.9% vs 29.7%, *p* < 0.001) in operated patients (Figs. 2b and 3c, respectively). After multivariable analysis, EOPC remained significantly associated with OS and CSS (Tables 3 and 4). For patients that did not undergo surgery, there were no significant differences in the 5-year OS (1.6% vs 1.4%; *p* = 0.874) or the 5-year CSS (1.8% vs 1.8%; *p* = 0.874), as shown in Figs. 2c and 3c.

**Table 3:** Multivariable Cox regression analysis for overall survival after propensity score matching

Variable	Hazard ratio	95% CI	*P* value
All patients, *N* = 6344			
EOPC vs LOPC, unadjusted	1.08	1.02–1.14	0.004
EOPC vs LOPC, adjusted^a^	1.07	1.01–1.13	0.015
Operated patients, *N* = 1738			
EOPC vs LOPC, unadjusted	1.34	1.20–1.49	< 0.001
EOPC vs LOPC, adjusted^b^	1.34	1.20–1.50	< 0.001
Non-operated patients, *N* = 4606			
EOPC vs LOPC, unadjusted	1.00	0.94–1.06	0.880
EOPC vs LOPC, adjusted^b^	1.01	0.95–1.07	0.824

^a^Adjusted for gender, tumor size, AJCC stage 7th edition, surgery, and chemotherapy

^b^Adjusted for tumor size, AJCC stage 7th edition, and chemotherapy

*CI*, confidence interval; *EOPC*, early-onset pancreatic cancer; *LOPC*, later-onset pancreatic cancer

**Table 4:** Multivariable Cox regression analysis for cancer-specific survival after propensity score matching

Variable	Hazard ratio	95% CI	*P* value
All patients, *N* = 6344			
EOPC vs LOPC, unadjusted	1.10	1.04–1.16	0.001
EOPC vs LOPC, adjusted^a^	1.09	1.03–1.15	0.003
Operated patients, *N* = 1738			
EOPC vs LOPC, unadjusted	1.40	1.25–1.57	< 0.001
EOPC vs LOPC, adjusted^b^	1.40	1.25–1.57	< 0.001
Non-operated patients, *N* = 4606			
EOPC vs LOPC, unadjusted	1.00	0.94–1.07	0.880
EOPC vs LOPC, adjusted^b^	1.02	0.95–1.08	0.613

^a^Adjusted for gender, tumor size, AJCC stage 7th edition, surgery, and chemotherapy

^b^Adjusted for tumor size, AJCC stage 7th edition, and chemotherapy

*CI*, confidence interval; *EOPC*, early-onset pancreatic cancer; *LOPC*, later-onset pancreatic cancer

## Discussion

To our knowledge, this is the largest study to date focusing on EOPC. We found that EOPC patients were diagnosed at a later stage and received more aggressive treatment. To control more comprehensively for biases associated with patient and treatment data, we stringently matched young and older patients using a propensity score matching method. In matched patients, inferior survival was observed for EOPC vs LOPC patients. Subgroup analyses showed that EOPC was associated with adverse survival in operated patients. However, for non-operated patients, the prognosis remained dismal without significant differences between the groups.

The relative frequency of EOPC varies between studies, from 4.4 to 18% [7–9, 16, 18, 19]. We report a relative frequency of 6.2%, which is within the range of previous studies. We found several differences in demographics and tumor characteristics between EOPC and LOPC. Patients with EOPC were more often male, which was also found in a previous study [16]. A possible explanation for the higher rate of EOPC in male patients may be smoking, which has been found to be a risk factor for EOPC [10]. We also show a higher rate of distant metastases in the EOPC group, as has been reported by others [8, 16].

Prior studies comparing outcomes between EOPC and LOPC have reported similar survival rates [7, 8, 16, 19]. However, most of these studies have not adjusted for the fact that the EOPC patients have more advanced disease at diagnosis and receive more active treatment as shown in the present study. The only previous study that adjusted for multiple co-variates was performed by Tingstedt et al. [8] who evaluated 33 EOPC patients and 33 matched controls using a case-control design. In the matched comparison, the survival tended to be shorter for the EOPC group, although not significant, likely due to the small cohort size.

In other cancer types, clinical and biomarker data suggest that early-onset cancers grow faster and are biologically more aggressive than later-onset cancers. For example, early-onset breast cancer has been reported to be more aggressive than later-onset breast cancer even after controlling for estrogen receptor and HER2 receptor status [20]. Also, in lung cancer, young age at disease onset seems to predict reduced survival [21].

The differences in clinical presentation and outcomes suggest that pancreatic cancer arising in young patients may be a distinct clinical entity. A study by Bergmann et al. [5] investigated tumor gene expression in 7 patients with EOPC. One interesting observation was that all tumors displayed a loss or significant reduction of SMAD4. SMAD4 is a tumor suppressor gene that is inactivated in 50–55% of pancreatic cancer patients. Inactivation of SMAD4 has been associated with a more aggressive phenotype of pancreatic cancer [22, 23]. Additionally, KRAS mutations were found in only 3 of 7 patients (42%) in the study by Bergmann et al. [5]. Traditionally, KRAS mutations are present in at least 90% of all patients. This suggests that at least a subgroup of EOPC patients may be genetically different from the common type of pancreatic cancer.

The use of a population-based study has the advantages of a large sample size. However, there are some limitations. The retrospective nature of the data introduces bias. This was partly overcome by using a propensity score–matched analysis in order to balance EOPC and LOPC groups based on several covariates. We were unable to adjust for histopathological differentiation due to the high degree of missing data. However, propensity score can only account for known imbalances; unknown confounders can still bias the results. Finally, the SEER radiation and chemotherapy data used in this study have their own limitations. The completeness of these variables and the potential biases associated with reasons for receiving or not receiving radiation or chemotherapy have been discussed in a previous publication [24].

## Conclusion

EOPC accounts for approximately 6% of pancreatic cancer patients. The diagnosis of EOPC is commonly made at a later stage and matched survival analysis suggests inferior outcomes compared with older patients. More research should be directed towards genomic and proteomic characterization of EOPC to investigate whether pancreatic cancer in young patients may be a distinct entity.
